# Attentional Orienting by Non-informative Cue Is Shaped via Reinforcement Learning

**DOI:** 10.3389/fpsyg.2019.02884

**Published:** 2020-01-15

**Authors:** Sang A. Cho, Yang Seok Cho

**Affiliations:** Department of Psychology, Korea University, Seoul, South Korea

**Keywords:** attentional orienting, spatial attention, value-driven attention, reinforcement learning, attentional bias

## Abstract

It has been demonstrated that a reward-associated stimulus feature captures attention involuntarily. The present study tested whether spatial attentional orienting is biased via reinforcement learning. Participants were to identify a target stimulus presented in one of two placeholders, preceded by a non-informative arrow cue at the center of the display. Importantly, reward was available when the target occurred at a location cued by a reward cue, defined as a specific color (experiments 1 and 3) or a color–direction combination (experiment 2). The attentional bias of the reward cue was significantly increased as trials progressed, resulting in a greater cue-validity effect for the reward cue than the no-reward cue. This attentional bias was still evident even when controlling for the possibility that the incentive salience of the reward cue color modulates the cue-validity effect (experiment 2) or when the reward was withdrawn after reinforcement learning (experiment 3). However, it disappeared when the reward was provided regardless of cue validity (experiment 4), implying that the reinforcement contingency between reward and attentional orienting is a critical determinant of reinforcement learning-based spatial attentional modulation. Our findings highlight that a spatial attentional bias is shaped by value via reinforcement learning.

## Introduction

Our cognition selects only a small amount of information from various sensory inputs in the environment at any given moment because of limits in our cognitive capability. Thus, our attention is allocated to a specific object, location, or feature to choose information for further processing. Visual attentional allocation is accomplished in two modes of attentional orienting. One depends on top–down factors, such as the task goal or performer’s intention, which is called endogenous attentional orienting. The other mode of attentional orienting is based on bottom–up factors, such as the physical salience of stimuli (spatial or temporal discontinuity), which is referred to as exogenous attentional orienting (Posner, 1980). Particularly, the exogenous mode of orienting is known to induce an involuntary capture based on stimulus features. Theories of an exogenous attentional-orienting mechanism have established what determinant is critical for the priority of involuntary attentional processing. For example, the contingent attentional capture account suggests that attention is allocated to a stimulus containing the target-defining feature for the task at hand (e.g., Folk et al., 1992). In contrast, the salience-driven attention theory insists that attentional deployment depends on the physical salience of stimuli (e.g., Theeuwes, 1992).

Recently, reward history has been suggested as another critical factor determining involuntary attentional orienting (see Wolfe and Horowitz, 2017; Failing and Theeuwes, 2018). In Anderson et al. (2011) experiment, participants were instructed to respond to the target inked in one of two colors in a search array that consisted of heterogeneously colored stimuli. Importantly, different amounts of monetary reward were given depending on the color of the target. In a subsequent visual-search task, in which the target was defined as a shape singleton, a stimulus inked in one of the two rewarded colors was presented as a distractor. They found that the distractor elicited attentional interference even though it was irrelevant and physically non-salient. Moreover, the amount of interference increased with the size of the associated value, independent of participants’ explicit knowledge about stimulus–reward relationships. This value-driven attentional capture (VDAC) has been consistently reported under other experimental manipulations using different types of stimulus features other than color, such as auditory stimuli (Anderson, 2015a), Gabor orientations (Laurent et al., 2015), neutral exogenous cues (Failing and Theeuwes, 2014), singleton distractors (Le Pelley et al., 2015), onset distractors (Munneke et al., 2016), or scenic pictures in a visual stream (Le Pelley et al., 2017). Importantly, these considerable amounts of evidence for VDAC by different stimulus features are commonly based on the relationships between features and their associated reward (Anderson, 2013, 2016; Bucker and Theeuwes, 2017). Specifically, attention is oriented toward the stimulus feature signaling reward because of its incentive salience, which is an emotional or motivational value of stimulus features, suggesting that when one feature of a stimulus predicts a significant outcome, such as reward, the feature becomes conspicuous as a conditioned stimulus (CS), resulting in attentional orienting toward the incentive-salient feature (MacLeod et al., 1986; Berridge and Robinson, 1998; Hickey et al., 2010; Hickey and Peelen, 2015). Specifically, Berridge and Robinson suggested that incentive salience is stimulus’ perceptual and motivational features that can capture attention. Thus, the findings of those studies on the relationship between attention and reward imply that the Pavlovian association between a stimulus feature and reward induces feature-based attentional allocation.

Critically, it has been suggested that VDAC is understood as a result of reinforcement learning. Importantly, while the attentional modulation by Pavlovian learning is dependent on the association between a stimulus with a reward-signaling feature and reward, the attentional modulation by reinforcement learning depends on habitual associations between spatial attentional orienting behaviors with a given specific stimulus feature and reward (see Balleine and O’doherty, 2010; Anderson, 2016; Bourgeois et al., 2016; Failing and Theeuwes, 2018). As attentional orienting toward a high reward target stimulus is reinforced repeatedly in a learning phase, this reinforced spatial attentional bias is persistently maintained even when this orienting response is unfavorable for the task performance in a subsequent test phase (e.g., Anderson et al., 2011). Recently, Le Pelley et al. (2015) tested this possibility by adopting a visual-search experiment in which the target was defined as a shape singleton, while a color singleton distractor signaled an obtainable reward. Participants were instructed to ignore a distractor for not only fast and accurate performance but also reward acquisition. They hypothesized that if reward reinforces the spatial attentional orienting toward the target stimulus in an instrumental manner, the target would be selected primarily, but a distractor signaling high reward would be more easily ignored than a distractor signaling low reward, resulting in a less interference effect for the distractor associated with high reward than one associated with low or no reward. Interestingly, however, they found significantly greater interference with the high-reward distractor than with the low-reward distractor, consistent with the findings of other studies with a similar method (Bucker et al., 2015; Pearson et al., 2015; Munneke et al., 2016). These results suggest that the value-driven attentional bias was obtained depending on the Pavlovian association between reward and stimulus feature rather than the reinforcement of spatial attentional orienting toward the target (see Bucker and Theeuwes, 2017). However, there is a possibility that these inconsistent findings regarding the influences of reinforcement learning on spatial attentional orienting was due to the interference from the competing influence of the feature-based attention based on the stimulus–reward association. For instance, in the experiment of Le Pelley et al. (2015), the reinforced orienting was measured as the attentional orienting toward the target when the reward-signaled distractor was presented in the target display. Thus, attentional orienting toward the target based on reinforcement learning could have competed with the attentional capture by the distractor based on the stimulus feature–reward association.

Although many previous studies have investigated whether value reinforces spatial attentional orienting (Harsay et al., 2011; Padmala and Pessoa, 2011; Chelazzi et al., 2013; Sawaki et al., 2015), the results were inconsistent across studies. Some studies reported no evidence for the reinforcement of attentional orienting when the orienting deteriorates search performance (Jiang et al., 2015; Won and Leber, 2016). For example, in the visual search experiments of Jiang et al. (2015) and Won and Leber (2016), although the target was randomly presented in each quadrant of the search display, a reward was provided only when it was presented at one of the four quadrants. If reward reinforced orienting to a particular quadrant, target search should have been more efficient in the rewarded quadrant than the other non-rewarded quadrants. They found that the reward effect on the spatial attentional bias to the rewarded quadrant was negligible or null. In contrast, some other studies found evidence for spatial attentional modulation by value reinforcement (Shomstein and Johnson, 2013; Chelazzi et al., 2014; Della Libera et al., 2017; Anderson and Kim, 2018a, b; McCoy and Theeuwes, 2018). For example, in the study of Chelazzi et al. (2014), when two targets were presented simultaneously in a visual search task, the target appearing at the locations previously associated with high reward was recognized more accurately than the other target appearing at the locations previously associated with low reward, which was maintained for several days after the end of learning. This suggests that participants allocated their attention to particular locations that had been imbued with high reward more frequently even though the targets were presented at random locations.

Of importance, these findings of the value modulation of spatial attention were compatible with the idea that reward reinforces a spatial attentional bias. That is, when reward was repeatedly delivered for attentional orienting toward a particular location, attention was deployed at the rewarded location. Importantly, these findings show that the spatial attentional bias was restricted to only a specific location. For example, in the study by Chelazzi et al. (2014), high rewarded locations were confined to only two among eight locations during reinforcement learning, while other six locations were associated with intermediate or low reward. In these cases, the spatial attentional bias is possibly understood as feature-based attentional modulation as the context-dependent phenomena resulted from Pavlovian-associative learning (or context specificity of Pavlovian learning). It indicated that the CS became conspicuous when the CS appeared within a particular context, in which Pavlovian conditioning has occurred (Lovibond et al., 1984; Bouton, 1993). Indeed, previous studies demonstrated that VDAC by a particular stimulus feature was modulated by the context information specified within a spatial context (Anderson, 2015b) or irrelevant background images in the target search display (Anderson, 2015c). Therefore, the Pavlovian learning can serve as possible explanations to these findings in that attentional allocation to the stimulus feature at a particular location was prioritized based on the association between reward and location.

The main purpose of the present study was to investigate whether spatial attentional orienting behaviors are implicitly strengthened by reinforcement learning. As reviewed above, previous literature demonstrated that attention is captured by a reward-signaling stimulus specified by a certain feature (e.g., Anderson et al., 2011; Le Pelley et al., 2015) or by a specific location (e.g., Chelazzi et al., 2014). Importantly, in these findings, two types of attentional modulation, feature-based attentional modulation based on Pavlovian learning and spatial attentional modulation based on reinforcement learning, were competed (e.g., Le Pelley et al., 2015) or at least confounded (e.g., Anderson et al., 2011; Chelazzi et al., 2014). Note that the attentional bias toward the reward-associated feature is a type of sign-tracking response, which refers to an approach response toward and engagement with the reward-predictive CS itself (Day and Carelli, 2007; Robinson and Flagel, 2009). Thus, the direction of the feature-based attentional modulation by reward is toward the location of the CS. In contrast, the reinforcement learning on spatial attentional orienting refers to the increased occurrence of attentional orienting as reward reinforces the orienting response. Critically, the direction of the reinforced attentional response is not determined by the location of the reward-signaling stimulus but by what attentional response is sufficiently reinforced. In other words, one of the important differences between the two types of attentional modulations is the direction of attentional orienting in that the orienting based on the Pavlovian association biases toward the location of the reward-signaling cue, whereas attentional orienting biases toward any location if the orienting response is strengthened based on reinforcement learning.

Therefore, the location of a reward-signaling feature and the direction of spatial attentional orienting should be separated to dissociate the spatial-based attentional modulation from the feature-based attentional modulation. For this, we adopted a non-informative central arrow in a modified version of the Posner cueing paradigm. Note that a non-informative symbolic cue (e.g., arrow) has been demonstrated to induce reflexive attentional orienting toward the arrow-pointed location, independent of exogenous and/or endogenous attentional control (Hommel et al., 2001; Tipples, 2002; Ristic and Kingstone, 2006, 2012). Thus, reward is expected to reinforce a particular spatial attentional response induced by a symbolic stimulus, which is understood as the reinforcement on a stimulus-response habit via reinforcement learning (Balleine and O’doherty, 2010). To reinforce the spatial attentional orienting selectively, reward delivery was contingent with the spatial attentional orienting toward the cued location, which was specified by the central arrow cue pointing to the left or right side of display. Specifically, participants were instructed to identify a target letter (L or T) presented at the left or right side of the display. Before the target presentation, the irrelevant non-informative arrow cue appeared at the center of display. There were two types of cues, reward and no reward, which were specified by the color of the arrow cue, such as red or green (experiments 1 and 3), or the combination of color and direction of the arrow cue, such as right-pointing red cue, left-pointing green cue, or vice versa (experiment 2). Importantly, reward was given only when the reward cue pointed validly to the location of the upcoming target, but no reward was provided when it was invalid or when the no-reward cue was presented regardless of its validity.

To observe a spatial attentional bias, the cue validity was measured with response time (RT) and percent error (PE) as indices of attentional orienting (Posner, 1980). Critically, if spatial attentional orienting is reinforced by reward, the reward cue would elicit a significantly greater cue-validity effect than the no-reward cue, even though these cues were irrelevant to the task. In addition, the reward modulation of the cue-validity effect would be gradually manifested as learning progresses, like the modulation of feature-based attentional allocation by Pavlovian learning (Anderson et al., 2014; Failing and Theeuwes, 2014; Le Pelley et al., 2015). Thus, to observe development of attentional modulation based on reinforcement learning, the cue-validity effect for the reward cue was compared to the no-reward cue in each block separately (experiments 1–4). If value reinforces an attentional orienting bias to the side corresponding to the direction of the reward cue, the cue-validity effect with the reward cue would increase as the block continued, whereas the effect for the no-reward cue would be not be changed across blocks. In addition, to examine whether the reinforced orienting persists after the reward was omitted, the cue-validity effect for each cue type was measured in a subsequent test phase after learning (experiments 3 and 4). Furthermore, to investigate whether the value-based spatial attentional bias was induced even without the reinforcement contingency between value and attentional orienting, reward was delivered regardless of cue validity in experiment 4. If the attentional bias to the cued location depends simply on the incentive salience of the cue based on the association between reward and cue feature, rather than the reinforcement contingency between reward and attentional orienting, the attentional bias would be greater for the reward cue than the no-reward cue.

## Experiment 1

Experiment 1 examined whether the reward reinforces spatial attentional orienting with a non-informative central cue. A leftward or rightward arrow, colored red or green, was used as a cue. One of the two cue types was associated with reward to reinforce attentional orienting to its directing location. Specifically, participants were instructed to search for the target letter at one of two placeholders. Before the presentation of the target letter, a red or green arrow pointing to the left or right placeholder was presented at the center of the display. To reinforce attentional orienting, reward points were given when the target was presented at the location pointed to by one color cue (reward cue), but no reward was given when the other color cue (no-reward cue) was presented, regardless of its validity.

If attentional orienting by a cue is reinforced by reward, the cue-validity effect would be larger for the reward cue than the no-reward cue. In addition, to monitor the progression of reinforcement learning on attentional orienting, the cue-validity effect for each cue type was monitored in each block. According to previous studies (Anderson et al., 2014; Failing and Theeuwes, 2014; Le Pelley et al., 2015), the influence of the reward association on performance would be relatively manifest in the latter blocks of trials.

### Materials and Methods

#### Participants

To determine proper sample sizes, we used G Power 3.1 (Faul et al., 2007). To estimate the difference of the cue-validity effect between the reward cue and the no-reward cue, we assessed the effect size from previous studies that tested the attentional orienting depending on the stimulus features (Lien et al., 2010) and the reward effect on attentional orienting (Failing and Theeuwes, 2014). The effect sizes in these studies (based on reported η*_*p*_*^2^ of the effects) ranged between 0.413 and 0.529. Accordingly, power analyses for within-sample analysis of variance (ANOVA) using a power of 0.95 and an alpha level of 0.05 yielded an appropriate sample size (*n*) between 14 and 21.

Sixteen undergraduate students (mean age = 23.4 years; 6 female and 10 male) were recruited from Korea University and provided with a monetary reward of KRW 6,000 (approximately 5 USD). To encourage their performance and earning of reward points, an incentive reward of KRW 1,000 (approximately 0.8 USD) was given when their response accuracy was above 80%. They were told that the sum of reward points should exceed a particular point for the additional incentive, but the amount was not explicitly stated to maintain their motivation to earn as many points as possible. All participants had normal or corrected-to-normal visual acuity and normal color vision by self-report. The current and following experiments were approved by the Institutional Review Board at Korea University (KU-IRB-16-138-A-1).

#### Apparatus

All experiments were programmed and presented using E-prime software (Version 2.0, Psychology Software Tools, Inc.). Stimuli were presented on a cathode ray tube monitor (17 in.) of a personal computer. Participants viewed the monitor from a distance of ∼60 cm in a dimly lit room. Responses were collected using a standard computer keyboard.

#### Stimuli

All stimuli were presented on a black background. Each trial consisted of a fixation display, a cue display, a target display, a feedback display, and a reward information display. In the fixation display, a white fixation cross (0.7° × 0.7° in visual angle) was presented at the center of the display, and two placeholders (2.1° × 2.1°) drawn with white lines (0.6°) were located at each side from the center of display (1.2°). In the cue display, an arrow stimulus (1.4° × 1.2°) colored red (*R* = 255, *G* = 0, *B* = 0; CIE color coordinates, *x* = 0.581, *y* = 0.346) or green (*R* = 0, *G* = 255, *B* = 0; CIE color coordinates, *x* = 0.285, *y* = 0.599) was presented at the center of the display, between the two placeholders, pointing to the left or right placeholder randomly (i.e., 50%). After another fixation display, the target display was presented; it consisted of a fixation cross at the center and two letters, each of which appeared in a placeholder. The target was defined as the letters L or T, and the non-target was a letter randomly selected from U, H, N, or E. Each character, in white Arial font, was 0.75° in width and 0.9° in height. The feedback display notified participants that their response was correct by presenting written feedback, “
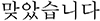
” (“Correct” in Korean), but for an incorrect response, a 1,000 Hz tone was sounded for 500 ms. The reward display informed participants on how many reward points they had earned on a given trial and the total amount of accumulated reward.

#### Procedure

Participants performed 16 practice trials followed by 512 experimental trials in 4 blocks of 128 trials. Each trial began with the fixation display for a random interval of 600, 800, or 1,000 ms. After the fixation display, the cue display was presented for 350 ms. The fixation display reappeared for 150 ms followed by the target display for 350 ms, and a blank display was presented until a response was made or for 1,000 ms. The feedback display appeared for 550 ms, and then, the reward information display was presented for 600 ms. After the delay of 200 ms, the next trial started.

**FIGURE 1:**
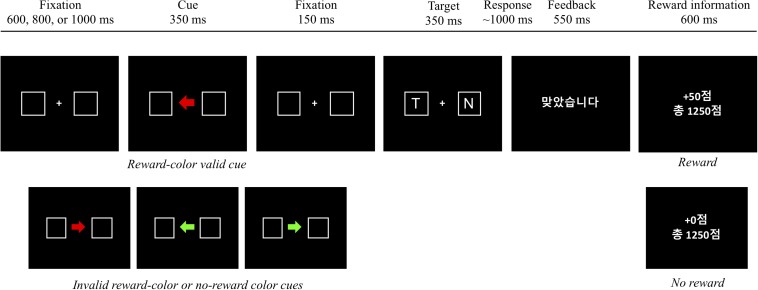
An example of a trial sequence in experiment 1.

Participants were instructed to identify the target letter, L or T, appearing inside one of the two placeholders and to ignore any other letter. Half of them were instructed to press the “f” key of a standard computer keyboard to the letter “L” and the “j” key to the “T,” and the other half were given the opposite target-key mappings. Because the direction of an arrow cue predicted the location of the target only 50% of the time – that is, randomly – it was a non-informative cue. Points were given (e.g., 0 or 50 points) as a reward, and participants were informed that the accumulated reward points should be maximized to obtain a monetary incentive. The participants were naive to when the reward was given, but they were instructed that the response had to be correct, at least, to earn reward scores. However, a reward score was obtainable for the correct response only when one of the two color arrow cues pointed to the target location validly (Figure 1). For example, 50 points were given when the participants made a correct response to a target that appeared in the placeholder to which a red arrow (reward cue) pointed, whereas no reward was given on the trials with the green arrow (no-reward cue). However, whenever a response was incorrect, 5 points were deducted from the total amount of accumulated reward, regardless of cue type and cue validity.

At the end of the experiment, participants were asked to complete an exit questionnaire to examine their explicit awareness about reward acquisition (e.g., Was there any specific type of trials for earning reward?) and how they perceived the predictability of the cue about the target location (e.g., How accurately did the cues predict the target location?) by open-ended questions. They did not see the questions before their participation.

#### Design

Cue type, cue color, cue direction, target location, and target letter were fully crossed and counterbalanced across participants. These types of trials were randomly presented. Mappings between target letter and response were also counterbalanced across participants.

### Results

Trials were excluded from analyses if RTs were shorter than 150 ms or longer than three standard deviations above the participants’ mean (2.06%), and only correct trials were included in RT analyses. Mean correct RTs and PEs were calculated for each participant as a function of block (first, second, third, or fourth block), cue type (reward cue or no-reward cue), and cue validity (valid or invalid). Repeated-measures ANOVAs were conducted on the mean RT and PE data, with those as within-subject factors.

#### RT

The overall mean RT was 508 ms. The main effect of cue type was significant, *F*(1, 15) = 6.66, *p* = 0.021, MSE = 228, $\eta_{p}^{2}$ = 0.308, showing that the mean RT was greater for reward cue (*M* = 511 ms) than for no-reward cue (*M* = 506 ms). The main effect of cue validity was also significant, *F*(1, 15) = 6.99, *p* = 0.018, MSE = 4,555, $\eta_{p}^{2}$ = 0.318, indicating that the mean RT was shorter on valid trials (*M* = 497 ms) than that on invalid trials (*M* = 519 ms). The interaction of block and cue type was significant, *F*(3, 45) = 3.38, *p* = 0.026, MSE = 259, η_*p*_^2^ = 0.184, suggesting that the mean RT for reward cue (*M*s = 516, 514, 508, and 505 ms in the first to fourth blocks, respectively) was greater than that for no-reward cue (*M*s = 522, 506, 499, and 495 ms in the first to fourth blocks, respectively) in the second and fourth blocks, but not in first and third blocks. More importantly, the interaction of cue type and cue validity was significant, *F*(1, 15) = 5.54, *p* = 0.033, MSE = 711, $\eta_{p}^{2}$ = 0.270. Separate analyses showed that a significant cue-validity effect was obtained for reward cue (30 ms), *F*(1, 15) = 8.69, *p* = 0.01, MSE = 3,345, $\eta_{p}^{2}$ = 0.367, whereas there was no significant effect for the no-reward cue (14 ms), *F*(1, 15) = 3.49, *p* = 0.081, MSE = 1,920. The interaction of block, cue type, and cue validity was not significant, *F*(1, 15) = 1.37, *p* = 0.26, MSE = 14, $\eta_{p}^{2}$ = 0.084, indicating that the interaction of cue type and cue validity in each block did not significantly differ.

#### PE

The overall PE was 1.65%. No main effect or interaction was significant, *F*s < 1.

#### Questionnaire

The responses from the exit questionnaire revealed that more than half of the participants (14 out of the 16) failed to notice the relationship between the reward availability and the cue type. All participants reported that the perceived predictability of the central arrow regarding the target location was random (e.g., 50%).

### Discussion

**FIGURE 2:**
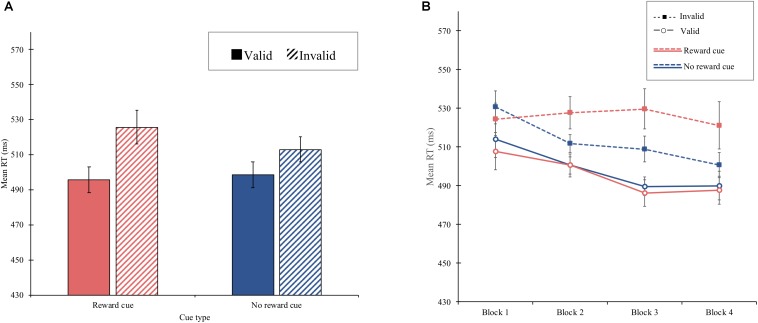
**(A)** Mean reaction time (in milliseconds) as a function of cue type and validity in experiment 1. **(B)** Mean reaction time (in milliseconds) as a function of block, cue type, and validity in experiment 1. Error bars ± within-subject standard error of the mean (Cousineau, 2005).

Experiment 1 demonstrated that reward reinforced spatial attentional orienting by a central cue, resulting in a significantly larger cue-validity effect for the reward cue (30 ms) than that for the no-reward cue (14 ms), shown in Figure 2A. Although these asymmetric cue-validity effects did not differ across blocks statistically, the amount of the cue-validity effect for the reward cue tended to increase from the first block (16 ms) to the fourth block (33 ms), whereas that for the no-reward cue did not (17 ms in the first block and 11 ms in the fourth block), shown in Figure 2B. It is possible that the attentional modulation by reward emerged quite early in learning so that the block effect failed to modulate the interaction between cue type and validity.

## Experiment 2

Experiment 1 showed that the central non-predictive cue strengthened a spatial attentional orienting on a basis of reinforcement learning. However, there is a possibility that this finding was simply resulted from the association between cue color and reward, rather than the reinforcement learning of spatial attentional orienting. Specifically, because the reward cue color became relatively incentive salient, it might have led attention toward its pointed lateral location. Thus, experiment 2 was conducted to examine whether the reward modulation of spatial attentional orienting obtained in experiment 1 resulted from the increased salience of the cue color rather than from the reinforcement learning of attentional orienting.

For this, the reward delivery depended on the combination of cue color and direction, rather than solely on cue color, in experiment 2. For example, reward was delivered for the correct response on valid trials only when the red arrow pointed to the left side or the green arrow pointed to the right side, whereas no reward was given for the correct response on valid trials when the cue had the opposite combinations of the features. Thus, since both colors were associated with the same amount of reward, the cue color itself did not signal reward. If the modulation of attentional orienting by reward found in experiment 1 was based simply on the association between cue color and reward, a similar amount of attentional bias would occur regardless of cue types. If the attentional bias to a specific location cued by a specific color of the central cue is reinforced by reward, the reward cues would induce a cue-validity effect, but the no-reward cues would not.

### Materials and Methods

#### Participants

A new group of 16 students (mean age = 24.4 years; 4 female and 14 male) from Korea University participated in experiment 2. As in experiment 1, they received a monetary reward of KRW 6,000 (approximately 5 USD), and if their accuracy was above 80%, they received another KRW 1,000 (approximately 0.8 USD) as an incentive.

#### Apparatus and Stimuli

Apparatus and stimuli in experiment 2 were identical to those used in experiment 1.

#### Procedure

**FIGURE 3:**
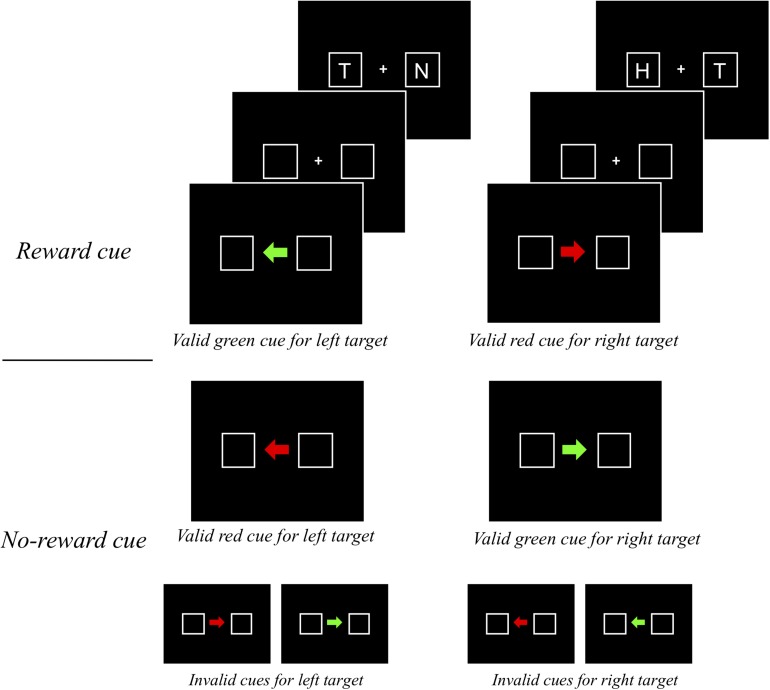
An example of a trial sequence in experiment 2.

The procedure was identical to that of experiment 1 except that reward did not depend on the color of the arrow cue. Specifically, reward points (e.g., 50 points) were offered for the correct response only on valid “reward cue” trials, which were defined as a function of cue color and direction of the arrow (Figure 3). For example, for half of the participants, the reward cue was a valid cue presented as a green arrow pointing to the left side of the display or a red arrow pointing to the right side, and for the other half, vice versa.

#### Design

The design was identical to that of experiment 1.

### Results

Regarding the trials, 1.80% were rejected from analyses by trimming the data using the same criteria used in experiment 1. Incorrect trials were excluded in RT analyses. Mean RT and PE were calculated for each participant as a function of block (first, second, third, or fourth block), cue type (reward cue or no-reward cue), and cue validity (valid or invalid). Repeated measures ANOVAs were conducted on the mean RT and PE data with those as within-subject factors.

#### RT

**FIGURE 4:**
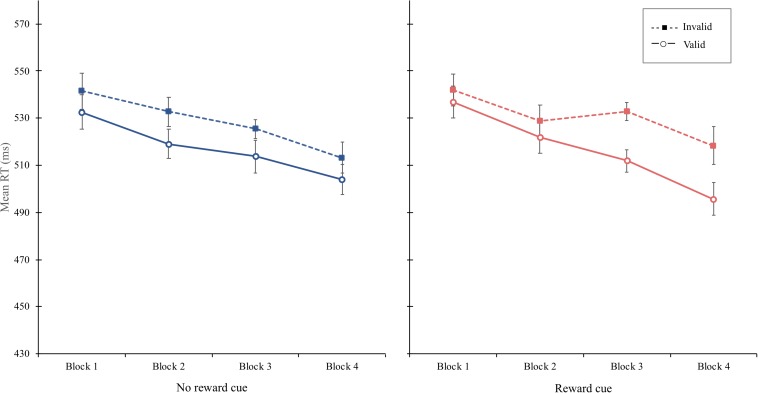
Mean reaction times (in milliseconds) as a function of block, cue type, and validity in experiment 2. Error bars ± 1 within-subject standard error of the mean (Cousineau, 2005).

The overall mean RT was 522 ms. The main effect of block was significant, *F*(3, 45) = 3.94, *p* = 0.014, MSE = 2,558, $\eta_{p}^{2}$ = 0.208. A significant main effect of cue validity was obtained, *F*(1, 15) = 10.95, *p* = 0.005, MSE = 894, $\eta_{p}^{2}$ = 0.422, with a shorter mean RT indicating faster responses on valid cue trials (*M* = 517 ms) than those on invalid trials (*M* = 529 ms). Although the interaction of cue type and cue validity was not significant, *F* < 1, the interaction of block, cue type, and cue validity was significant, *F*(3, 45) = 3.13, *p* = 0.034, MSE = 122, $\eta_{p}^{2}$ = 0.173. Simple interaction effect analyses revealed that the interaction of cue type and cue validity was not significant in the first, second, *F*s < 1, and third blocks, *F*(1, 45) = 2.49, *p* = 0.121, MSE = 122, $\eta_{p}^{2}$ = 0.210, but significant in the fourth block, *F*(1, 45) = 4.07, *p* = 0.049, MSE = 122, $\eta_{p}^{2}$ = 0.302. Simple main effect analyses indicate that the cue-validity effect was significantly greater for the reward cue (23 ms), *F*(1, 15) = 18.42, *p* < 0.001, MSE = 222, $\eta_{p}^{2}$ = 0.55, than the no-reward cue (9 ms), *F*(1, 15) = 3.59, *p* = 0.08, MSE = 192, $\eta_{p}^{2}$ = 0.193, in the fourth block. Additional analyses showed that the significant interaction of block and cue validity was obtain for reward cue, *F*(3, 45) = 3.57, *p* = 0.021, MSE = 189, $\eta_{p}^{2}$ = 0.192, not for the no-reward cue, *F* < 1. This finding implied that as blocks progressed, the amount of the attentional bias by reward cue significantly increased, whereas that by the no-reward cue was constant (Figure 4).

#### PE

The overall PE was 1.81%. The main effect of cue validity was significant, *F*(1, 15) = 6.90, *p* = 0.019, MSE = 0.0003, $\eta_{p}^{2}$ = 0.3151, indicating that more errors were committed on invalid trials (2.10%) than those on valid trials (1.50%). Any other main effect or interaction did not reach significance, *F*s < 1.

#### Questionnaire

The exit questionnaire revealed that all participants were not aware of the relationship between the reward and cue type. In addition, the perceived cue predictability about the target location was random (e.g., 50%).

### Discussion

Experiment 2 shows that even when the reward cue was defined as a function of both cue color and cue direction, the attentional bias to the side to which the reward cue pointed was gradually manifested as blocks progressed (Figure 4). The magnitude of the cue-validity effect increased about five times (5–23 ms) from the first to the last block only for the reward cue, whereas it was nearly constant for the no-reward cue (9–9 ms in the first and last block, respectively), implying that through sufficient reinforcement learning, participants biased their attention to the side compatible with the direction of the reward cues. Importantly, this reward modulation was not simply attributed to the association between cue color and reward because the reward was not signaled by the cue color itself. Rather, reward-contingent orienting was strengthened as learning progressed even when the feature of reward cue was defined as a combination between color and direction.

However, there are some other alternative possibilities to explain the spatial attentional bias corresponding to the direction of the reward cue. That is, as the attentional modulation by value was obtained during reinforcement learning in experiments 1 and 2, participants might have been more likely to orient their attention toward the location cued by the reward features in a given trial if they had received reward with the same cue features in previous trials, which is referred to as a reward-modulated priming effect (Della Libera and Chelazzi, 2006; Hickey et al., 2010). In addition, although the questionnaire results showed that participants failed to explicitly notice the relationship between reward and cue features, it may be a premature conclusion that the underlying processes were implicit. When considering previous studies showing that the power of the questionnaire was not sufficiently sensitive to detect all relevant explicit knowledge (Lovibond and Shanks, 2002; Vadillo et al., 2016), that participants might have oriented their attention toward the cued location strategically in an implicit way to earn more reward when the reward-signaling cue was presented (Lovibond and Shanks, 2002; Vadillo et al., 2016). Thus, to assess the reinforcement influence on attentional orienting when no reward is provided after learning, we employed an unrewarded independent task to examine the attentional modulation by value after learning in experiment 3.

## Experiment 3

Experiment 3 was aimed to test whether the attentional bias toward the cued location by the reward cue is consistently obtained even when the reward is omitted after learning. For this purpose, participants were asked to perform two different visual search tasks in two distinct phases: learning and test. Specifically, in the learning phase, a visual search task, which was identical to the task used in experiment 1, was employed to induce a spatial attentional bias by reinforcing attentional orienting to the location indicated by the reward cue. Importantly, after the learning phase, participants performed another search task in the test phase in which they were instructed to identify the orientation of a target line preceded by one of the non-informative central cues that had been presented as the reward or no-reward cue in the learning phase. Critically, no reward was provided regardless of cue type in the test phase.

Previous studies have shown that value-based attentional modulation persistently occurs in the unrewarded test phase (Anderson et al., 2011; Chelazzi et al., 2014; Le Pelley et al., 2015). Therefore, if a spatial attentional bias was caused by the reward-modulated priming and/or the strategic attentional control, the attentional modulation by value would be observed in the learning phase but not in the test phase. However, if the attentional modulation by value was based on the reinforcement learning effect on spatial attention, the asymmetrical attentional bias would occur depending on the cue type not solely in the learning phase but also in the test phase. Since there is a possibility that the reinforcement learning effect would be extinct due to the omission of reward in the test phase, the cue-validity effects for both cue types were measured in two blocks.

### Materials and Methods

#### Participants

Sixteen new students (mean age = 22.4 years; 8 female and 8 male) from Korea University were recruited as participants in experiment 3.

#### Apparatus

Apparatus was same to that used in experiments 1 and 2.

#### Stimuli

All stimuli in the learning phase were identical to those used in experiment 1. In the test phase, the target display consisted of the fixation cross at the center of the display and the target stimulus defined as a horizontal or vertical line segment inside one of two placeholders. No reward information display was used in the test phase. The other displays in the test phase were identical to those in the learning phase.

#### Procedure

The procedure of the learning phase was identical to that of experiment 1, with the following exceptions. The learning phase consisted of 4 blocks of 96 trials. The amount of reward per a trial changed from 50 in the previous experiments to 75 and won (unit of KRW), instead of point(s), was used as the reward denomination.

**FIGURE 5:**
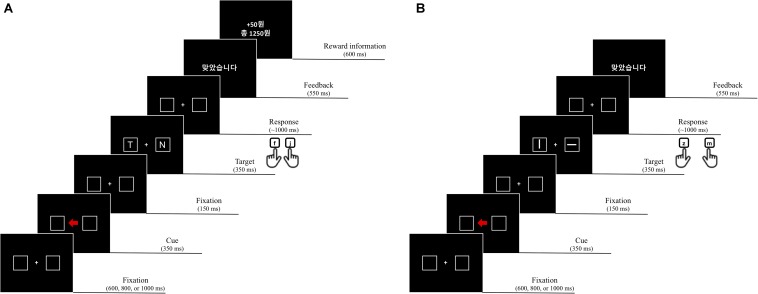
Examples of a trial sequence of the learning phase **(A)** and the test phase **(B)** in experiment 3.

After performing the search task in the learning phase, participants were instructed to perform another search task, which was test phase. The test phase consisted of 2 blocks of 96 trials. Participants were to identify a target line orientation (horizontal or vertical) appearing inside one of the two placeholders by pressing the response keys (e.g., “z” key to the horizontal line and “m” key to the vertical line) while ignoring a non-target line, which was 45° tilted clockwise or counterclockwise, on the other side (Figure 5).

#### Design

The design was identical to that of experiment 1.

### Results

With the same criteria used in the previous experiments, 1.76% of trials in the learning phase and 1.60% of trials in the test phase were removed from analyses. Only correct trials were included in RT analyses. Mean correct RT and PE in the learning phase were calculated for each participant as a function of block (first, second, third, and fourth block), cue type (reward cue or no-reward cue), and cue validity (valid or invalid). Mean correct RT and PE in the test phase were calculated as a function of block (first and second block), cue type (reward or no-reward cue), and cue validity (valid or invalid). Repeated measures ANOVAs were conducted on the mean RT and PE data, with those variables as within-subject factors.

#### Learning Phase

**FIGURE 6:**
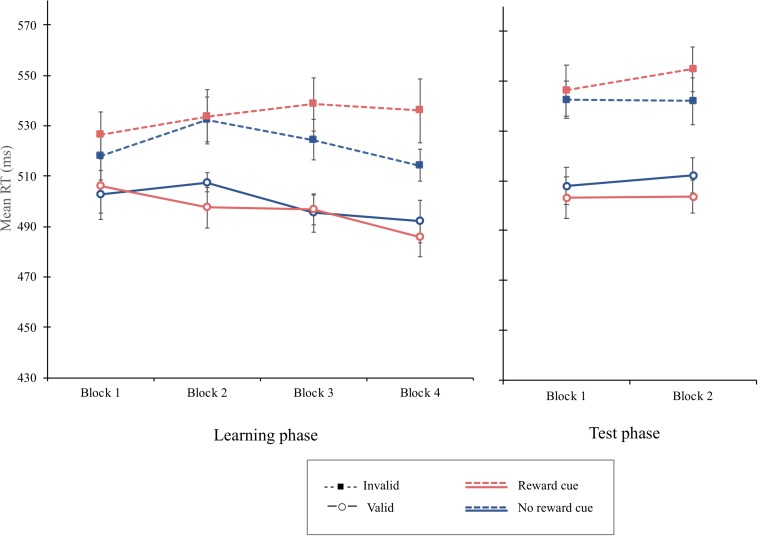
Mean reaction times (in milliseconds) as a function of block, cue type, and validity in the learning phase (left) and the test phase (right) of experiment 3. Error bars ± 1 within-subject standard error of the mean (Cousineau, 2005).

The overall mean RT was 512 ms. The main effect of cue type failed to reach the significant level, *F*(1, 15) = 4.54, *p* = 0.0501, MSE = 246, indicating that the mean RT for the reward cue (*M* = 515 ms) tends to be greater than for the no-reward cue (*M* = 511 ms). The main effect of cue validity was significant, *F*(1, 15) = 10.06, *p* = 0.006, MSE = 5,713, $\eta_{p}^{2}$ = 0.402. The mean RT was shorter on valid trials (*M* = 498 ms) than invalid trials (*M* = 528 ms). Importantly, the interaction of cue type and cue validity was significant, *F*(1, 15) = 4.67, *p* = 0.047, MSE = 690, $\eta_{p}^{2}$ = 238, indicating that the cue-validity effect was greater when the reward cue was presented (37 ms), *F*(1, 15) = 9.64, *p* = 0.007, MSE = 456, $\eta_{p}^{2}$ = 0.391, than when the no-reward cue was (22 ms), *F*(1, 15) = 9.10, *p* = 0.009, MSE = 1,841, $\eta_{p}^{2}$ = 0.378. Moreover, the three-way interaction of block, cue type, and cue validity was significant, *F*(3, 45) = 2.99, *p* = 0.041, MSE = 129, $\eta_{p}^{2}$ = 0.166 (Figure 6). Simple interaction effect analyses showed that the interaction of cue type and cue validity was not significant in the first block, *F* < 1, but failed to reach significance in the second block, *F*(1, 45) = 3.92, *p* = 0.054, MSE = 129, $\eta_{p}^{2}$ = 0.304, and significant in the third block, *F*(1, 45) = 5.16, *p* = 0.028, MSE = 129, $\eta_{p}^{2}$ = 0.365. Critically, cue type interacted significantly with cue validity in the fourth block, *F*(1, 45) = 24.2, *p* < 0.001, MSE = 129, $\eta_{p}^{2}$ = 0.72. Simple main effect analyses demonstrated that a significant cue-validity effect was obtained when the reward cue was presented, *F*(1, 15) = 8.76, *p* = 0.0097, MSE = 2292, $\eta_{p}^{2}$ = 0.37, but not when the no-reward cue was presented, *F*(1, 15) = 3.37, *p* = 0.086, MSE = 1,164, $\eta_{p}^{2}$ = 0.18, in the fourth block.

The overall PE was 2.12%. The main effect of cue validity was significant, *F*(1, 15) = 13.36, *p* = 0.0023, MSE = 0.001, $\eta_{p}^{2}$ = 0.4711, indicating that, with a greater PE, more errors were committed on invalid trials (2.85%) than on valid trials (1.41%). Importantly, the interaction of cue type and cue validity was significant, *F*(1, 15) = 5.13, *p* = 0.038, MSE = 0006, $\eta_{p}^{2}$ = 0.2549. Separate analyses indicated that the magnitude of the cue-validity effect was larger when the reward cue was presented (2.10%), *F*(1, 15) = 13.42, *p* = 0.0023, MSE = 0.0011, $\eta_{p}^{2}$ = 0.4721, than when the no-reward cue was presented (0.77%), *F*(1, 15) = 3.94, *p* = 0.066, MSE = 0.0005 (see Table 1).

**TABLE 1:** Percent errors (standard deviation in parentheses) in experiments 1–4 as a function of cue type and cue validity.

		**Cue type**	
		**No-reward cue**	**Reward cue**
Experiment 1	Invalid	1.89 (2.50)	2.01 (3.38)
	Valid	1.37 (2.16)	1.35 (2.12)
	*Cue-validity effect*	0.52	0.66
Experiment 2	Invalid	1.92 (2.64)	2.32 (3.48)
	Valid	1.67 (2.79)	1.34 (2.30)
	*Cue-validity effect*	0.24	0.98
Experiment 3	Invalid	2.29 (3.72)	3.41 (4.28)
	Valid	1.52 (3.20)	1.31 (2.87)
	*Cue-validity effect*	0.78	2.10
Experiment 4	Invalid	4.97 (6.62)	5.01 (6.40)
	Valid	4.03 (5.96)	3.60 (4.82)
	*Cue-validity effect*	0.93	1.41

#### Test Phase

The overall mean RT was 526 ms. The main effect of cue validity was significant, *F*(1, 15) = 21.43, *p* = 0.0003, MSE = 2,334, $\eta_{p}^{2}$ = 0.5883, with a greater mean RT indicating that the mean RT was significantly greater on invalid trials (*M* = 546 ms) than that on valid trials (*M* = 507 ms). Critically, the interaction of cue type and cue validity was significant, *F*(1, 15) = 7.21, *p* = 0.016, MSE = 246, $\eta_{p}^{2}$ = 0.3248, indicating that a greater cue-validity effect was obtained when the reward cue was presented (47 ms), *F*(1, 15) = 22.9, *p* = 0.0002, MSE = 1,542, $\eta_{p}^{2}$ = 0.6042, than when the no-reward cue was presented (32 ms), *F*(1, 15) = 15.88, *p* = 0.0012, MSE = 1,038, $\eta_{p}^{2}$ = 0.5142. The three-way interaction of block, cue type, and cue validity was not significant, *F* < 1, implying that the value modulation on attentional orienting was not extinct. The overall PE was 3.47%. No other main effect or interaction was significant in the PE data, *F*s < 1.

### Discussion

In the learning phase, as in the previous experiments, the value-based attentional modulation was obtained, which gradually increased as learning progressed. More importantly, as in the learning phase, RT and PE data in the test phase indicated that the attentional bias was consistently greater when the reward cue was presented than when the no-reward cue was, although the reward was no longer delivered. The attentional bias obtained in the test phase is an S–R habit, which refers to the learned tendency to repeat a specific response contingent on features of context (Wood and Neal, 2007) because attentional orienting to the location primed by the reward cue was no longer goal-directed behavior (Balleine and O’doherty, 2010). It implies that the value-modulated attentional bias appeared without the reward-modulated priming and the motivation for the reward acquisition. Thus, the continued spatial attentional biases guided by the reward cue in the test phase supports the modulatory effect of reinforcement learning on spatial attention, independent of the reward-modulated priming effect and strategic attentional control.

## Experiment 4

Even though the attentional orienting induced by a non-informative arrow cue was found to be strengthened by reward in the previous experiments, this value-based attentional modulation could have been due to the increased incentive salience of the reward cue based on the Pavlovian association between the cue color and reward. To examine whether the obtained value-based attentional modulations were as a result of reinforcement learning or Pavlovian learning, the two types of learning effects should be separated. In the previous experiments, reward was available only when the target was presented at the location cued by the reward cue to reinforce the attentional orienting toward the cued location. Note that this pairing between reward and attentional orienting guided by the reward cue, which refers to the reinforcement contingency, is critical for reinforcement learning because this contingency between reward and behavior increases the probability of the reoccurrence of the behavior as instrumental conditioning (Skinner, 1969, 2014).

Thus, experiment 4 was conducted to examine whether the value-based attentional bias occurs without the reinforcement contingency between reward and attentional orienting toward the location cued by the reward cue. The methods used in experiment 3 were employed in experiment 4 with an exception that reward was delivered on invalid trials, as well as on valid cue trials, when the reward cue was presented. Hence, participants were expected to receive a reward with 50% probability when the reward cue was presented regardless of its validity. Therefore, experiments 3 and 4 were identical in the probabilistic associative relationship (e.g., 50%) between the reward cue and the reward delivery, but they differed in the reinforcement contingency, such as 100% probability for the valid reward cue in experiment 3 and 50% for the valid reward cue and 50% for the invalid reward cue in experiment 4.

Importantly, the possibility of the modulation of spatial attentional orienting simply by the association between reward and feature still remained to some degree in the previous experiments. In particular, if reward increased the incentive salience of the reward cue defined as a function of cue color and direction selectively, such as the red cue directing the left side of the display, the incentive-salient arrow might have increased an orienting response to the cued location. Importantly, the reward cue color was associated with reward at 50% probability in experiment 4, as in all previous experiments. Therefore, if the Pavlovian association between reward and cue feature is sufficient to increase an attentional bias regardless of the reinforcement learning on spatial attentional orienting, the asymmetrical attentional bias depending on cue type would occur. In contrast, if the reinforcement contingency between reward and particular attentional orienting underlying the reinforcement learning was a determinant of spatial attentional biases, neither the difference in the attentional modulation by value between cue types nor the increment of the spatial attentional bias by the reward cue would occur.

### Materials and Methods

#### Participants

A different group of 16 participants (mean age = 22.1 years, 11 female and 5 male) were recruited from the same pool, and they received monetary rewards applied to the rules used in experiment 3.

#### Apparatus and Stimuli

Apparatus and stimuli were identical to those in experiment 3.

#### Procedure

The procedure was identical to that of experiment 3, except that reward did not depend on the cue validity of the arrow cue in the learning phase. For instance, reward (e.g., 75 won) was provided for the correct response on “reward cue” trials, which were simply defined as a cue color, regardless of cue validity. For example, for half of the participants, the reward cue was a green arrow cues, which signaled reward at 50% probability, regardless of whether it was valid or invalid, and vice versa for the other participants.

### Results

With identical criteria applied as in the previous experiments, 1.40% of trials in the learning phase and 1.63% of trials in the test phase were excluded from the RT and PE data with the identical criteria applied in the previous experiments.

#### Learning Phase

**FIGURE 7:**
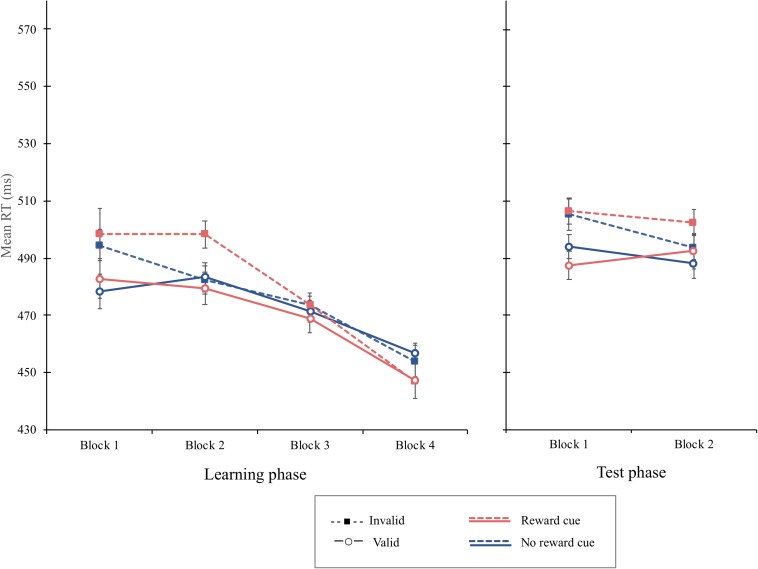
Mean reaction times (in milliseconds) as a function of block, cue type, and validity in the learning phase (left) and the test phase (right) of experiment 4. Error bars ± 1 within-subject standard error of the mean (Cousineau, 2005).

The overall mean RT was 473 ms. The main effect of cue validity was significant, *F*(1, 15) = 4.64, *p* = 0.047, MSE = 614, $\eta_{p}^{2}$ = 0.236, showing that the mean RT was shorter when the cue was valid (*M* = 471 ms) than when it was invalid (*M* = 478 ms). The interaction of cue type and cue validity was not significant, *F*(1, 15) = 1.94, *p* = 0.184, MSE = 291. The cue-validity effect for reward cue (9 ms), *F*(1, 15) = 4.36, *p* = 0.054, MSE = 683, was not different from that of the no-reward cue (3 ms), *F*(1, 15) = 1.98, *p* = 0.180, MSE = 222. Interestingly, the interaction of block and cue validity was significant, *F*(3, 45) = 3.56, *p* = 0.021, MSE = 255, $\eta_{p}^{2}$ = 0.192. Simple main effect analyses revealed that the cue-validity effect was significant in the first block, *F*(1, 15) = 12.03, *p* = 0.0034, MSE = 337, $\eta_{p}^{2}$ = 0.4450, but not in the second, *F*(1, 15) = 2.53, *p* = 0.1323, MSE = 506, $\eta_{p}^{2}$ = 0.144, third, or fourth blocks, *F*s < 1, indicating that the attentional bias elicited by irrelevant central cues disappeared after the second block. The interaction of block, cue type, and cue validity was not significant, *F*(3, 35) = 2.03, *p* = 0.123, MSE = 174, $\eta_{p}^{2}$ = 0.119, suggesting that the interaction of block and cue validity was not different as a function of cue types (Figure 7). An additional analysis using Bayes factors was conducted to provide a more robust analysis supporting the lack of interaction. The Bayes Factor for the interaction of cue type and cue validity in the learning phase was computed to compare the likelihoods of the null hypothesis to the alternative hypothesis that there is a difference in the cue-validity effect depending on cue type. No significant interaction between cue type and cue validity was supported by a Bayes factor (BF_10_) of 0.309, which suggests substantial evidence for the null hypothesis (JASP Team, 2019).

The overall PE was 4.41%. There was no significant main or interaction effect in the PE data, *F*s < 1.

#### Test Phase

The overall mean RT was 495 ms. Although the main effect of cue validity was significant, *F*(1, 15) = 12.94, *p* = 0.0026, MSE = 322, $\eta_{p}^{2}$ = 0.4631, the interaction of cue type and cue validity was not significant, *F*(1, 15) = 1.58, *p* = 0.227, MSE = 181, indicating that, even though the mean RT of valid trials (*M* = 491 ms) was shorter than that of the invalid trials (*M* = 502 ms), no significant difference in the cue-validity effect was found between the reward cue (14 ms) and the no-reward cue (9 ms) (Figure 7). The overall PE was 6.45%. The main effect of block was significant, *F*(1, 15) = 12.06, *p* = 0.0034, MSE = 0.0014, $\eta_{p}^{2}$ = 0.4457, indicating with a that more errors were committed in the first block (7.59%) than those in the second block (5.28%). The cue-validity effect failed to reach the significant level, *F*(1, 15) = 4.03, *p* = 0.063, MSE = 0.003. PE was 5.47 and 7.41% on valid and invalid trials, respectively. No other main or interaction term was significant in the PE data, *F*s < 1.

### Discussion

No significant difference in the amount of the spatial attentional bias was found between the reward and no-reward cues when no contingency between reward and attentional orienting toward the location cued by the reward cue existed. In addition, the attentional orienting by the reward cue did not increase but rather decreased with blocks. Thus, even though the reward cue was paired with the reward at 50% probability, as in experiment 3, the evidence for the spatial attentional modulation by value reinforcement was not found. Therefore, this discrepancy in the results of attentional bias across experiments supports the incentive-salient cue based on the Pavlovian association between reward and cue feature is insufficient to replicate the spatial attentional bias to the cued location obtained in the previous experiments. Instead, it suggests that the reinforcement contingency between reward and attentional orienting is a crucial factor for the attentional modulation by value to occur.

## General Discussion

The present study demonstrated that reward strengthens spatial attentional orienting via reinforcement learning. Specifically, in experiment 1, the cue-validity effect was significantly greater with a non-predictive central reward cue than the no-reward cue, indicating that spatial attention was reinforced to be oriented to the side corresponding to the direction of the reward cue. However, there was a possibility that the increased incentive salience of the reward cue based on the Pavlovian association between the irrelevant cue color and reward might have led participants to orient their attention to the location corresponding to the direction of the reward color cue. This possibility was controlled in experiment 2 wherein both colors were associated with the same amount of reward by designating the reward cue as a function of cue color and cue direction. Consequently, the cue-validity effect became more evident for the reward cue than for the no-reward cue as learning progressed. In experiment 3, we tested whether the spatial attentional bias caused by the reward cue would persist even when reward was omitted in a subsequent test phase. As in the previous experiments, a greater attentional bias for the reward cue was obtained than for the no-reward cue during the learning phase. More importantly, it remained evident in the test phase as well. These results imply that the value modulation on attention via reinforcement learning persists even in an unrewarded context, independent of the reward-modulated priming effect from the prior rewarded trial to a given trial or intentional attentional shifting toward the location pointed by the reward cue due to the motivation to receive more reward. However, although the findings of experiment 2 indicate that the attentional biases elicited by the reward cue in experiments 1 and 3 were not solely due to the incentive salience of cue color, a possibility still remained that the incentive salience of the conjunctive features of color and direction modulated attentional orienting, regardless of reinforcement learning on attentional orienting. Hence, experiment 4 examined whether the value-based spatial attentional modulations occur without the reinforcement contingency between reward and attentional orienting related to cue validity but solely with the Pavlovian association between reward and cue color. Importantly, the reward-signaling color cue failed to induce a greater attentional bias than the no-reward cue and rather the cue validity for the reward cue was diminished as the task progressed.

Indeed, the further analysis using Bayes factors in the learning phase of experiment 4 supported that attentional orienting was not enhanced by the reward cue relative to the no-reward cue. It implies that the reward-orienting contingency underlying reinforcement learning was essential for the spatial attentional bias. Moreover, instead of the spatial attentional bias elicited by the reward cue, we found the cancelation of attentional orienting for all cue during the learning phase of experiment 4. When considered that the arrow cue induces reflexive attentional orienting (Hommel et al., 2001; Tipples, 2002; Ristic and Kingstone, 2006, 2012), the nullification of the cue-validity effect implies that when the central cue signaled reward without the reinforcement contingency between reward and orienting, the attentional bias was obtained in a manner of the sign-tracking response which is an approach response toward the location of the CS due to the Pavlovian stimulus–reward association, resulting in the disappearance of the cue-validity effect by the central arrow cue.

The key manipulation of the present study is that the reward is contingent with spatial attentional orienting as a cognitive behavior using a central cue. Note that the cue used in all experiments was a central arrow cue, which has been typically used in an endogenous cueing paradigm to study voluntary attentional orienting (Posner, 1980), which depends on the cue “informativeness” about how much the cue validly predicts the location of upcoming targets (Jonides, 1981; Vossel et al., 2006; Saban et al., 2017). However, the cues used in the present study are not compatible with the requisition of endogenous cues because the arrow cue had no information about the upcoming target location. Rather, it has been suggested that the symbolic cue, such as arrow and eye gaze, elicits attentional orienting in an automatic fashion. According to previous research, this reflective attentional orienting is independent of the volitional attentional orienting by endogenous cues (Hommel et al., 2001; Ristic and Kingstone, 2006) or the exogenous orienting by a periphery onset stimulus (Ristic and Kingstone, 2012). Indeed, the main effect of cue validity was significant in all experiment of the present study, indicating that the central arrow cue generally guided attention despite of the lack of predictive information.

### The Difficulty of Learning and Attentional Modulation

Basically, the associative structures underlying the instrumental conditioning consist of three elements, which are descriptive stimulus, instrumental response, and outcome (Colwill, 1994; Gámez and Rosas, 2007). The descriptive stimulus, which refers to a stimulus that informs a performer of the availability or property of outcome, modulates the association between response and outcome (Rescorla, 1991). In this view, the color (experiments 1 and 3) or the combination of color and direction (experiment 2) informed the availability of reward, and thus, performers had to learn the relationship between cue features and reward. Hence, it was more difficult to learn the features of discriminative stimuli when the reward-signaling feature was defined as a function of two features, color and direction, than when it was defined as a single feature, color. Indeed, the cue-validity effect was greater for the reward cue than the no-reward cue only in the last block in experiment 2, while the greater cue-validity effect for the reward cue than the no-reward cue was evident regardless of blocks in experiment 1.

In addition, the difficulty of learning for the relationship between instrumental response and outcome is possibly considered as a reason for the inconsistency of evidence for the spatial attentional modulation by value across studies. For example, in the studies reporting no evidence for the spatial attentional bias by value, the search array consisted of 12 (Jiang et al., 2015) or 16 (Won and Leber, 2016) stimuli in the possible regions of a 10 × 10 matrix, giving 100 possible target locations in these display settings, whereas a reward was provided only when the target was presented in one quadrant, which consisted of 25 different locations in the T-among-L tasks. In contrast, the studies reporting evidence of value modulation on spatial attention adopted visual search tasks, in which the number of potential target locations was relatively small, such as four (Anderson and Kim, 2018a, b), eight (Chelazzi et al., 2014; Della Libera et al., 2017), and two in the present study. Therefore, reward was more likely to reinforce the attentional orienting toward a particular location selectively when the reinforcement contingency between instrumental response and reward is relatively less difficult.

According to de Wit and Dickinson (2009), instrumental responses should be based on the knowledge of the relationships between responses and their outcomes and the outcome should be desirable. Importantly, although the results of the exit questionnaire indicated that participants did not have explicit knowledge of the relationships, the reinforced attentional responses might have depended primarily on their implicit knowledge between attentional orienting toward the location cued by the reward cue and reward.

### Learning Mechanism Underlying Attention Modulations

In a recent review, Wolfe and Horowitz (2017) concluded that reward acts as a modulator to increase the effectiveness of feature-based attention because the stimulus feature, such as color, is essentially attention guiding. That is, reward plays a role in increasing the salience of stimulus features so that attention is deployed on this incentive-salient feature (Berridge and Robinson, 1998; Hickey et al., 2010; Hickey and Peelen, 2015). However, our findings did not result simply from the value reinforcement on feature-based attentional allocation because the reward-associated feature was presented at the center of the display, but the reinforced attentional bias was toward the non-salient cued placeholder. Rather, when the central cue signaled the reward without any contingency between particular orienting and reward, the incentive-salient feature captured and held attention, resulting in the lack of attentional orienting despite its symbolic shape (see experiment 4).

Beyond the feature-based attentional modulation by value, some studies revealed that spatial attentional bias toward a specific location is elicited by reward and even persists in an unrewarded context (Chelazzi et al., 2014; Della Libera et al., 2017; Anderson and Kim, 2018a, b). For example, Chelazzi et al. (2014) showed that a target was identified more accurately when it was placed at high reward-associated locations than at low reward-associated locations even for several days after learning. This long-term reward modulation on spatial attention reflects a plastic alteration on the spatial priority maps, which indicates the behavioral salience of specific locations. In contrast, the attentional biases found in the present study are not location specific in that attentional orienting was increased toward the location pointed by the reward cue in a given trial compared to the no-reward cue. Therefore, the attentional modulation based on reinforcement learning depends on whether the attentional orienting by cue direction is reinforced, independent of location.

Taken together, unlike previous studies that showed the modulation of either feature- or spatial-based attention by reward, the attentional bias by reward obtained in this study depended on the interplay of feature- and spatial-based attentional processing. In all the present study’s experiments, since the reward availability depends on the feature of the central cue, the effect of reinforcement learning was discriminatively obtained depending on the cue feature. In other words, the cue feature is related to the occurrence of the value-based attentional bias. However, as verified in experiment 4, the spatial attentional bias did not occur simply when the stimulus feature signaling the reward was presented. That is, since a reward was delivered only when the reward cue predicted the target location validly, it was contingent upon the attentional orienting to the cued location, resulting in a spatial attentional bias. Therefore, the reinforcement contingency causes the spatial attention to be involuntarily biased toward cued location.

This relationship between cue features and spatial attentional biases is closely involved with the result of Pavlovian-Instrumental Transfer (PIT), which refers to a phenomenon that a Pavlovian stimulus that is associated with a reward evokes or increases the instrumental responses associated with the same reward (Rescorla and Solomon, 1967; Kruse et al., 1983; Talmi et al., 2008; Corbit and Balleine, 2005; Nadler et al., 2011; see the review by Holmes et al., 2010; Cartoni et al., 2016). The typical PIT paradigm consists of a Pavlovian conditioning phase in which a cue stimulus, such as a tone, is associated with a reward, such as a food pellet, and an instrumental conditioning phase, in which the same reward is provided for an instrumental response, such as lever pressing. After these two phases, the probability of the instrumental response increases when the Pavlovian stimulus is presented. Intriguingly, this PIT fits well to explain the reward modulation of attentional orienting found in this study, in which reward was predicted by the cue feature (i.e., Pavlovian stimulus), whereas attentional orienting to the location guided by the cue was an instrumentally learned response.

**FIGURE 8:**
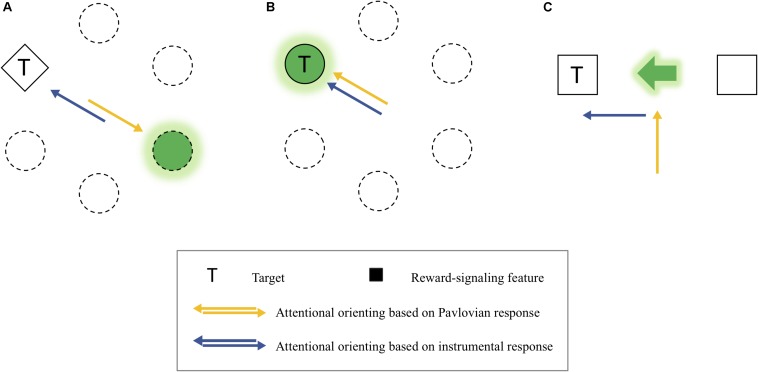
**(A)** An example of the competitive relationship between the Pavlovian and instrumental response-based orienting. **(B)** An example of the confounding relationship between them. **(C)** An example of dissociated attentional orienting based on the Pavlovian and instrumental response.

In the PIT experiments, a Pavlovian stimulus evokes a Pavlovian response, as well as an instrumental response. That is, the Pavlovian response refers to performers’ approach behavior toward the CS itself (sign-tracking) or the site of reward delivery (goal-tracking) when a Pavlovian stimulus is presented (Schwartz, 1976; Overmier and Lawry, 1979; Tomie, 1996; Holmes et al., 2010). For example, if a rat learns to press a lever (i.e., instrumental response) when a light turns on (i.e., CS), the rat approaches the CS or the site of reward provision (e.g., a reward magazine) instead of pressing the lever, indicating that the Pavlovian response interferes with the instrumental response. Importantly, this competition between the two types of responses provides insight into some previous inconsistent findings on the instrumental modulation of attention by reward (see Figure 8). More specifically, depending on different levels of the competition between the Pavlovian and instrumental responses, different patterns of the instrumental modulation of attention by reward were obtained. On the one hand, in the experiments of Le Pelley et al. (2015) in which reward was signaled by a color singleton distractor, a reward was given when participants successfully attended to a shape singleton target. Because the attentional capture by the distractor (i.e., Pavlovian response) was opposite to the attentional orienting to the target (i.e., instrumental response), the competition between the two responses seemed to be intense (Figure 8A). Consistent with other similar studies (Bucker et al., 2015; Pearson et al., 2015; Munneke et al., 2016), they failed to find the instrumentally conditioned attentional modulation but found the attentional capture by the reward-associated distractor, which is a Pavlovian response. In other studies, in which a specific target feature (e.g., Anderson et al., 2011) or a target at a particular location (e.g., Chelazzi et al., 2014; Della Libera et al., 2017; Anderson and Kim, 2018a, b) predicted a reward, the level of the competition between the two types of response was almost null because attentional orienting to the reward-signaling stimulus belongs to not only the Pavlovian response but also the instrumental response during learning (Figure 8B). In these cases, however, the influences of the two types of responses were confounded, so it is unclear whether the modulation of attention by value was caused by the Pavlovian or the instrumental conditioning.

It is important to note that the behavioral effects of the two types of responses were dissociated in the present study (Figure 8C). The instrumentally reinforced response (spatial attentional orienting to the cued location) was the spatial attentional bias measured by the magnitude of the cue-validity effect, which depended on the reinforcement contingency (experiments 1–3). Importantly, here this instrumentally reinforced response was independent of the Pavlovian responses that are attending to the center of display where both the reward-cue feature (i.e., sign-tracking response) and the reward points (i.e., goal-tracking response) were presented, which emerged as attentional holding at the center when the reinforcement contingency was excluded (experiment 4). Thus, as in the present study, it is possible to consider the dissociation between the Pavlovian stimulus and instrumental responses underlying spatial-based attention to understand the value-reinforced attentional bias found in the present study.

## Conclusion

Skinner (1953) noted, “Operant conditioning shapes behavior as a sculptor shapes a lump of clay” (1953, p. 91). In the same vein, the findings of the present study emphasized value as a crucial motivator that triggers our attention, which is understood as one of cognitive behaviors, beyond the role as an attractor that draws attention as previously reported, consistent with Pavlovian learning theory. Indeed, through repeated examinations, we demonstrated that value can shape the spatial attentional allocation instrumentally even when it is irrelevant for achieving the top–down goal. In conclusion, our attentional system is not employed only to achieve the current task goal but also to operate for value, as a long-term goal of our motivational system.

## Data Availability Statement

The datasets generated for this study are available on request to the corresponding author.

## Ethics Statement

The studies involving human participants were reviewed and approved by The Institutional Review Board at Korea University. The patients/participants provided their written informed consent to participate in this study.

## Author Contributions

SC and YC designed the study. SC acquired and analyzed the data. Both authors contributed to the interpretation of results and writing of the manuscript.

## Conflict of Interest

The authors declare that the research was conducted in the absence of any commercial or financial relationships that could be construed as a potential conflict of interest.
